# Death Rate of Various Cities

**Published:** 1874-03

**Authors:** 


Death-rate of Various Cities.—Dr. Charles P. Russell gives a tabulated statement of the mortality of the various States of the Union, from which we borrow the following regarding the death-rates of various cities: The highest death-rate in 1872 was exhibited by Memphis, where the deaths were 46.6 in each 1,000 inhabitants. Other cities followed in this order: Savannah, 39.2; Vicksburg, 36.5; Troy, 34; Hoboken, 32.9; New York, 32.7; Newark, 31.6; New Orleans, 30.6; Boston, 30.5. The rate for Philadelphia was only 26.1; Brooklyn, 28.1; St. Louis, 20.1; Chicago, 27.6; Baltimore, 25.1; Cincinnati, 20.5; San Francisco, 17.2. This compares not unfavorable with the mortuary statistics of British cities, where the lowest rate was 21.4; that of London, Bombay, and Calcutta, show only 26.2 and 25, respectively. The highest known death-rate prevailed in Valparaiso, Chili, 66.9. *From Popular Science Monthly, for February.*

